# Editorial: Reviews in pathology of infectious diseases

**DOI:** 10.3389/fvets.2024.1435676

**Published:** 2024-06-14

**Authors:** Jaime Gomez-Laguna, Francisco J. Pallares, Francisco J. Salguero

**Affiliations:** ^1^Department of Anatomy, Comparative Pathology, and Toxicology, Pathology and Immunology Group (UCO-PIG), UIC Zoonosis y Enfermedades Emergentes ENZOEM, University of Córdoba, International Excellence Agrifood Campus ‘CeiA3', Córdoba, Spain; ^2^Department of Pathology, UK Health Security Agency (UKHSA), Porton Down, Salisbury, United Kingdom; ^3^School of Biosciences and Medicine, University of Surrey, Guildford, United Kingdom

**Keywords:** pathology, infectiuos diseases, review, emerging disease, animal model

The importance of infectious diseases in animals, especially zoonotic diseases, has increased dramatically in recent years with the COVID-19 pandemic, the emergence and/or re-emergence of vector-borne diseases, avian influenza, Mpox infection and many others, together with the persistence of endemic diseases such as PRRS in pigs or tuberculosis in multiple hosts (1–3). This Research Topic focuses on the pathology of infectious diseases that affect animals and humans, emphasizing the value of animal disease models to combat them.

Hunter et al. reviewed the pathological aspects of pulmonary tuberculosis in animal models, including rodents, guinea pigs, non-human primates, rabbits, ruminants, and zebrafish. They produced a comprehensive description of the lesions induced by *Mycobacterium tuberculosis* complex (MTBC) in the lungs of infected animals and the use of these models in research. Moreover, they described the pulmonary lesions observed in human tuberculosis, as a comparative exercise with a “One Health” approach.

Larenas-Muñoz et al. described the characterization of the pathological lesions present in the guinea pig model of *Mycobacterium tuberculosis* infection, using a combination of classic histopathology, immunohistochemistry and digital image analysis. The detailed description of the evolution of granulomatous lesions in this important pre-clinical model of tuberculosis, will be very valuable in evaluating antimicrobial therapies and vaccines that may eventually progress to clinical phases.

Layton et al. described the impact of stress and anesthesia on animal models of infectious disease analyzing the most recent scientific evidence on these two variables and reviewing the strategies and technologies used to monitor animal models during infectious diseases.

Ruedas-Torres et al. reviewed the host-pathogen interactions during Porcine Reproductive and Respiratory Syndrome (PRRS) infection in pigs. They described the main lesions observed in the pulmonary aspect of this disease: interstitial pneumonia, suppurative bronchopneumonia and proliferative and necrotising pneumonia, emphasizing the differences observed between strains of different virulences.

Sorensen et al. described the effects of water temperature on Piscine orthoreovirus genotype 3 (PRV-3) infection in rainbow trout (*Oncorhynchus mykiss*), showing that low water temperatures allow for higher PRV-3 replication, which is associated with more severe heart pathology and increased expression of important antiviral genes.

Rodrigues da Silva et al. reviewed the porcine circovirus 3 infection in pigs, an increasing problem for the pork industry worldwide, describing the epidemiology, clinical manifestations, pathological co-infections and diagnostics.

Dorfelt et al. presented a comprehensive retrospective study of tetanus in dogs, a severe neurological disease that can also affect humans, caused by *Clostridium tetani*, and associated with high mortality.

Naseem et al. described the pathology and pathogenesis of the cutaneous lesions observed in beef cattle associated with buffalo fly (*Haematobia irritans exigua*) infestation, including the secondary infections that may be associated with this parasitic disease.

Flores-Velazquez et al. reviewed the pathogenesis, and host-parasite interactions in fasciolosis, another parasitic disease caused by infestation with *Fasciola hepatica*, also focusing on the current work being carried out to develop a vaccine against this disease, which suffers from an increasing problem of anthelmintic resistance.

## Author contributions

JG-L: Writing – original draft, Writing – review & editing. FP: Writing – original draft, Writing – review & editing. FS: Writing – original draft, Writing – review & editing.
